# Maize yield in smallholder agriculture system—An approach integrating socio-economic and crop management factors

**DOI:** 10.1371/journal.pone.0229100

**Published:** 2020-02-24

**Authors:** Sudarshan Dutta, Somsubhra Chakraborty, Rupak Goswami, Hirak Banerjee, Kaushik Majumdar, Bin Li, M. L. Jat

**Affiliations:** 1 African Plant Nutrition Institute, Benguérir, Morocco; 2 Agricultural and Food Engineering Department, IIT Kharagpur, Kolkata, India; 3 IRDM Faculty Centre, RKMVERI, Kolkata, India; 4 Regional Research Station (CSZ), BCKV, Kakdwip, India; 5 Department of Experimental Statistics, Louisiana State University, Baton Rouge, Louisiana, United States of America; 6 International Maize and Wheat Improvement Center (CIMMYT), New Delhi, India; University of Education Lahore, Pakistan, PAKISTAN

## Abstract

Yield gaps of maize (*Zea mays* L.) in the smallholder farms of eastern India are outcomes of a complex interplay of climatic variations, soil fertility gradients, socio-economic factors, and differential management intensities. Several machine learning approaches were used in this study to investigate the relative influences of multiple biophysical, socio-economic, and crop management features in determining maize yield variability using several machine learning approaches. Soil fertility status was assessed in 180 farms and paired with the surveyed data on maize yield, socio-economic conditions, and agronomic management. The C&RT relative variable importance plot identified farm size, total labor, soil factors, seed rate, fertilizer, and organic manure as influential factors. Among the three approaches compared for classifying maize yield, the artificial neural network (ANN) yielded the least (25%) misclassification on validation samples. The random forest partial dependence plots revealed a positive association between farm size and maize productivity. Nonlinear support vector machine boundary analysis for the eight top important variables revealed complex interactions underpinning maize yield response. Notably, farm size and total labor synergistically increased maize yield. Future research integrating these algorithms with empirical crop growth models and crop simulation models for ex-ante yield estimations could result in further improvement.

## 1 Introduction

The Sustainable Development Goals to eradicate poverty (Goal 1), hunger (Goal 2) and improve human health and well-being (Goal 3) [1] will require a 60% to 110% increase in global agricultural production. FAO’s State of the World series [2], and IFPRI’s visionary 2050 policy documents have identified food security as the global concern of the 21^st^ Century. Bridging the large yield gaps in smallholder farms of Asia and Africa, with significant regional and interpersonal variations, is necessary to reduce global food insecurity [3, 4]. The intensively cultivated eastern part of India [5] is characterized by smallholder farms [6]. Inherently, the smallholder farming systems function under a broad array of biophysical, climatic, and socio-economic settings, and their improvement is often hindered by inadequate access to land, fertile soil, capital, and labor [7, 8]. The interactions among these factors affect resource use efficacy and the ability to produce optimal yield. Tittonell et al. [9] concluded that biophysical and socio-economic factors, linked to diverse local climates, soil types, access to markets, and socio-cultural and ethnic characteristics govern soil fertility and crop yield variation. In fact, yield-gap analyses have recently taken adequate account of smallholder heterogeneity to identify local/regional factors of yield variation [10, 11, 12, 13]. Understanding these determinants of yield variability in smallholder systems is important to formulate informed policies to close the yield gap for major food crops.

Maize (*Zea mays* L.) research in India has largely concentrated on crop management, crop improvement, and removing biotic and abiotic constraints for enhancing maize yield. However, how these factors function within the structural, biophysical, and socio-economic contexts of farming has been less explored [14, 15, 16]; therefore, assessing the relative significances of soil and crop management, socio-economic and structural factors is important for targeted site-specific management interventions [17, 18].

Methods of measuring yield variability and productivity gaps frequently utilize experimental results obtained at the local level [19] or at the regional/global level [20], with scant attention given to the inherent variability in farm conditions. The large variability in crop growth and yield in time and space challenges the accuracy of existing models [21, 22]. An assessment of the impacts of climatic, biophysical, management, and socio-economic determinants is necessary to understand the causes of yield variability in farm fields [23]; however, our understanding of the interactions between these factors for predicting crop yield is still limited. While one group of researchers used classical statistical methods, such as correlation, regression, and cluster analysis to analyze yield variability [24, 25], others preferred different process-based models to study on-farm yield gaps [26, 27]. While the relative superiority of the process-based crop growth models over empirical models is well established, the increased demands of technological complexity and robust calibration–verification measures are the main limiting factors for their broader application, particularly in smallholder farms of developing countries that lack financial and technical capacities [28]. Given that the empirical crop growth models play a crucial role in identifying the hidden structure of the crop growth process, the most deterministic models sometimes heavily rely on the former, i.e. process-based models [29]. Investigating multiple interactions among the outcome and the explanatory variables often demands adaptive and non-parametric multivariate analyses, due to their ability to negotiate non-linear relationships, thus overcoming the limitations of Euclidian distance-based general linear models. Data collected by field surveys are a mix of continuous, discrete, and categorical variables, and are often found to be highly skewed. To handle such complexities, classification and regression tree (C&RT) analysis has recently been employed by several researchers to categorize relatively homogeneous observations in terms of target and explanatory variables [14, 30]. Further, techniques like support vector machine (SVM) and artificial neural network (ANN) have been efficiently used to identify the complex and non-linear relationships between target and predictor variables.

This study is a continuation of the work by Banerjee et al. [14], and investigates the underlying multifaceted links between maize yield and biophysical, socio-economic, and crop management factors by applying several multivariate machine learning approaches. We intend to put forward a compelling case to the agricultural scientists and policymakers for using these approaches to explain maize yield in smallholder farms. The specific objectives of this communication are: (i) to identify the key socio-economic, crop management, and biophysical factors for predicting maize yield; (ii) to understand the underlying relationship between the abovementioned factors for determining maize yield in small farms of two agroecological zones of eastern India; and (iii) to compare the relative efficiency of different machine learning approaches to classify maize yield variability.

## 2 Materials and methods

### 2.1 Site description

The study was conducted in two districts of West Bengal, India: Malda in the ‘Old Alluvial’ and Bankura in the ‘Red and Lateritic’ agro-climatic zones. Together, these two zones cover an area of 10,615 km^2^. The climate in Malda is hot and humid in the summer, with an average annual rainfall of 1453 mm. The climate of Bankura is drier with an average annual rainfall of 1400 mm [14]. The population density is 446 and 881 inhabitant km^-2^ for Bankura and Malda, respectively [31]. During the survey of secondary information, Banerjee et al [14] recorded several features of farming in the area that are relevant for this study. First, the districts reflect different altitudes, soil types, ethnic groups, and land use patterns. Second, the total, net sown area in the studied districts ranged from 260,000 ha to 345,000 ha. The cropping intensity ranged from 164 to 183%. Farms in both the districts are predominantly small and marginal with landholding of less than 1.0 ha. Three distinct crop seasons can be found in both the districts: *pre-kharif* (March-May), *kharif* (June-October), and *rabi* (November-February). Maize has emerged as an important crop in both Malda (during *pre-kharif*, *kharif* and *rabi* seasons) and Bankura (during *kharif* season). Malda produced 20 thousand t of maize grain from 8620 ha, greater than the acreage (172 hectares) and productivity (2.3 t ha^-1^) of Bankura [14, 31].

### 2.2 Farm surveys and soil laboratory characterization

The study was conducted on private agricultural land, with permission from the owners. Ramakrishna Mission Vivekananda University’s ethics committee approved the locations by involving farmer participants before the study began. Two *Blocks* (smaller administrative units of community development comprised of village clusters) with the highest maize growing areas were selected for the survey from 15 blocks of Malda and 22 blocks of Bankura districts (Table 1). Three villages in each of the selected blocks were chosen in consultation with the Program Coordinators of the Farm Science Centre (a First Line Extension agency of Indian Council of Agricultural Research), the deputy director of agriculture, local non-governmental organizations, and progressive farmers. The villages with high maize acreage under the identified maize growing seasons, were selected. Maize-growing farmers in the villages (30 farmers from each village) were then selected for the detailed survey through systematic sampling [14]. Pre-survey interactions with farmers were carried out along with the survey of maize fields to understand the existing status of maize cultivation. This was followed up with a day-long stakeholder consultation, leading to the formulation of a structured interview schedule (see S1 File) [14]. The pre-tested questionnaires were used in the structured interviews with the owners of 180 farms (90 farms per district). These were coupled with visits to the maize fields of each household.

**Table 1:** Study locations in West Bengal, India.

District	Block	Village	Latitude (N) (In Degree Decimal)	Longitude (E) (In Degree Decimal)	Closely identified soil series	Classification^a^
Bankura	Chatna	Dalpur	23.34	86.91	Gangajalghati	Fine-loamy, mixed, hyperthermic, Typic Ustochrepts
		Kendua	23.37	86.96		
		Suyarabagra	23.49	86.96		
	Gangajal Ghati	Bamundiha	23.49	87.23	Gangajalghati	Fine-loamy, mixed, hyperthermic, Typic Ustochrepts
		Kayamati	23.39	87.05		
		Shuyabasa	23.64	87.08		
Malda	English Bazar	Madia	25.19	88.15	Alinagar	Coarse-loamy, mixed, hyperthermic, Typic Ustifluvents
		Naraharipur	25.11	88.08		
		Niyamatpur	25.05	88.19		
	Gazole	Bhabanipur	25.45	88.28	Dakshin Harishchandrapur	Fine, mixed, hyperthermic, Aeric Endoaquepts
		Durgapur	25.52	88.32		
		Uttar Maldanga	25.35	88.21		

^a^NBSS&LUP (2001)

A total of 180 composite surface (0–60 cm) soil samples were collected from an equivalent number of fields prior to maize planting. Each composite sample was a mixture of eight subsamples from each field. The samples were air-dried, ground, and passed through a 2 mm sieve. Soils were analyzed for particle size [32], saturated paste pH [33], salinity [33], total organic carbon [34], available S [35], available K, available P [36,37], and available N [38].

We collected maize yield data from farmer’s reports and validated 20% of data (n = 36) with allometric models defined by Tittonell et al. [39], which fell inside the 95% confidence interval. Explanatory variables were grouped under socio-economic, management, structural, and soil-related variables. The measurement of these variables is given in Table 2.

**Table 2:** Explanatory variables used in the C&RT analysis.

Variables	Description
*Socio-economic*	
Farming experience	Number of years the farm family is engaged in crop cultivation; measured in years;
Ethnic origin	The ethnic identity of the farm household as per the stipulation of Government of India; Categorised as–Non-tribe– 1 and Scheduled Tribe– 2
Socio-Economic Status Class	Measured by modified Kuppuswamy’s socio-economic scale (Kumar et al., 2012)
Household size	Number of members in a farm family who share food from a single source; Absolute number of members in a family
Members of the family working in own farm	Number of members in a farm family who work within the farm completely or partially for sustaining livelihood
Non-farm income	Income (Indian Rupees) of the farm family in a year from non-farm sources
Wage earning	Whether the farm family earns a wage from working in others’ farms; Yes = 1; Otherwise = 0
Ownership of cultivable land	Whether the farm family has own land, which is lawfully recorded; Yes = 1; Otherwise = 0
Farm size	Size of the homestead and owned cultivable land (ha) recorded lawfully
Topography of land	Whether the land is ‘level’ or ‘undulated’ as perceived by the respondent; Level-1, Undulated = 0
*Management Factors*	
Leguminous crop in the cropping sequence	Whether at least one leguminous crop is grown on the plot where the maize was grown; Yes = 1; Otherwise = 0
Constraint in Irrigation	Whether irrigation is a constraint in non-monsoon months; Yes = 1; Otherwise = 0
Spacing R-R	Spacing between two rows of Maize plant (cm)
Spacing P-P	Spacing between two Maize plants within a row (cm)
Seed type	Genetic nature of seed used in maize cultivation; Traditional-1; Hybrid-2
Seed rate	Amount of maize seed used in cultivation plot (t ha^-1^)
Organic manure	Amount of organic sources of plant nutrient used in maize cultivation plot (t ha^-1^)
Fertilizer	Amount of inorganic sources of plant nutrient used in maize cultivation plot (Kg ha^-1^)
Insecticide	Amount of active ingredient of plant protection chemicals used in maize cultivation plot (g ha^-1^)
Total labour	Total family and hired labour used for all operations related to maize cultivation (man-hour ha^-1^)
The severity of soil problem	Perceived strength of soil problem; No– 0; Light– 1; Moderate– 2
*General and Structural variables*	
Agro-ecological region	Bankura District = 1, Malda = 2
Distance to input	Physical distance (km) of farms to farm input market
Distance to market	Physical distance (km) of farms to farm output market
*Soil variables*	
	Principal component scores of the soil wet chemistry data (PC1 and PC2)
	Principal component scores of the soil spectral data (SPC1 and S PC2)

### 2.3 Soil spectral characterization

Traditionally, laboratory-based, routine, soil physicochemical analyses have been the basis for our perception of soil quality and function; however, there is a pressing need for the development of fast and cost-effective methodologies for soil analyses in precision agriculture. Hyperspectral diffuse reflectance spectroscopy, a rapid and non-destructive approach, has been used as an alternative soil analytical approach for the last two decades [40]. We scanned 180 soil samples using a portable ASD FieldSpec^®^ spectroradiometer (Analytical Spectral Devices, CO, USA) [see S2 File (SM) for more details on spectral analysis and spectral modeling]. To reduce the dimensionality of the spectral data (10-nm interval) in subsequent modeling analysis, principal component analysis (PCA) was performed which selected the first two PCs (SPC1, SPC 2) that summarized 90% of the total spectral variation. Additionally, PCA performed on soil wet chemistry indices selected the first two PCs (PC1 and PC2) that together explained 88% of the total variability. A significant correlation was found between clay and organic carbon (p<0.0001). The PC1 explained 54% of total variation while PC2 explained 34% of the variation. Subsequently, all four abovementioned PCs (SPC1, SPC2, PC1, and PC2) were incorporated in the “Soil Factors” in Table 2 as an alternative to using soil wet chemistry data and soil spectral data to classify maize yield.

### 2.4 Multivariate modeling

#### 2.4.1 Classification of maize yield

In the present study, we first used a C&RT algorithm known for predicting quantitative or classifying categorical targets by recursively dividing the dataset [41]. The C&RT analysis was done by SPM software (Salford Systems, San Diego, CA, USA). Maize yield was used as a target variable, and socio-economic, management, and soil factors (topography along with spectral and wet chemistry PCs) were used as explanatory variables (Table 2) in this study. Among other multivariate models, random forest (RF), support vector machine (SVM), and artificial neural network (ANN) analyses were conducted [42, 43, 44] [See S2 File SM for more details]. Maize yield (t ha^-1^) was converted *a priori* into discrete classes [1^st^ quartile (Q_1_), 2^nd^ quartile (Q_2_), 3^rd^ Quartile (Q_3_), and 4^th^ quartile (Q_4_)] for classification purpose. The ANN was run in the WEKA data mining package. We optimized parameters of ANN via ‘CVParameterSelection’ module. Note that RF, SVM, and ANN were applied on the whole dataset and further applied on a split of data (135 training 75% and 44 test 25%).

#### 2.4.2 Predicting maize yield by RF regression

After establishing the influencing variables by the abovementioned classification algorithms, the RF regression was used to predict the Maize yield using the whole dataset with full cross-validation. The coefficient of determination (R^2^), cross-validation RMSE (RMSEcv), residual prediction deviation (RPD), and bias were used for judging model predictability.

## 3. Results

### 3.1 Maize yield and soil characterization

**Fig 1:**
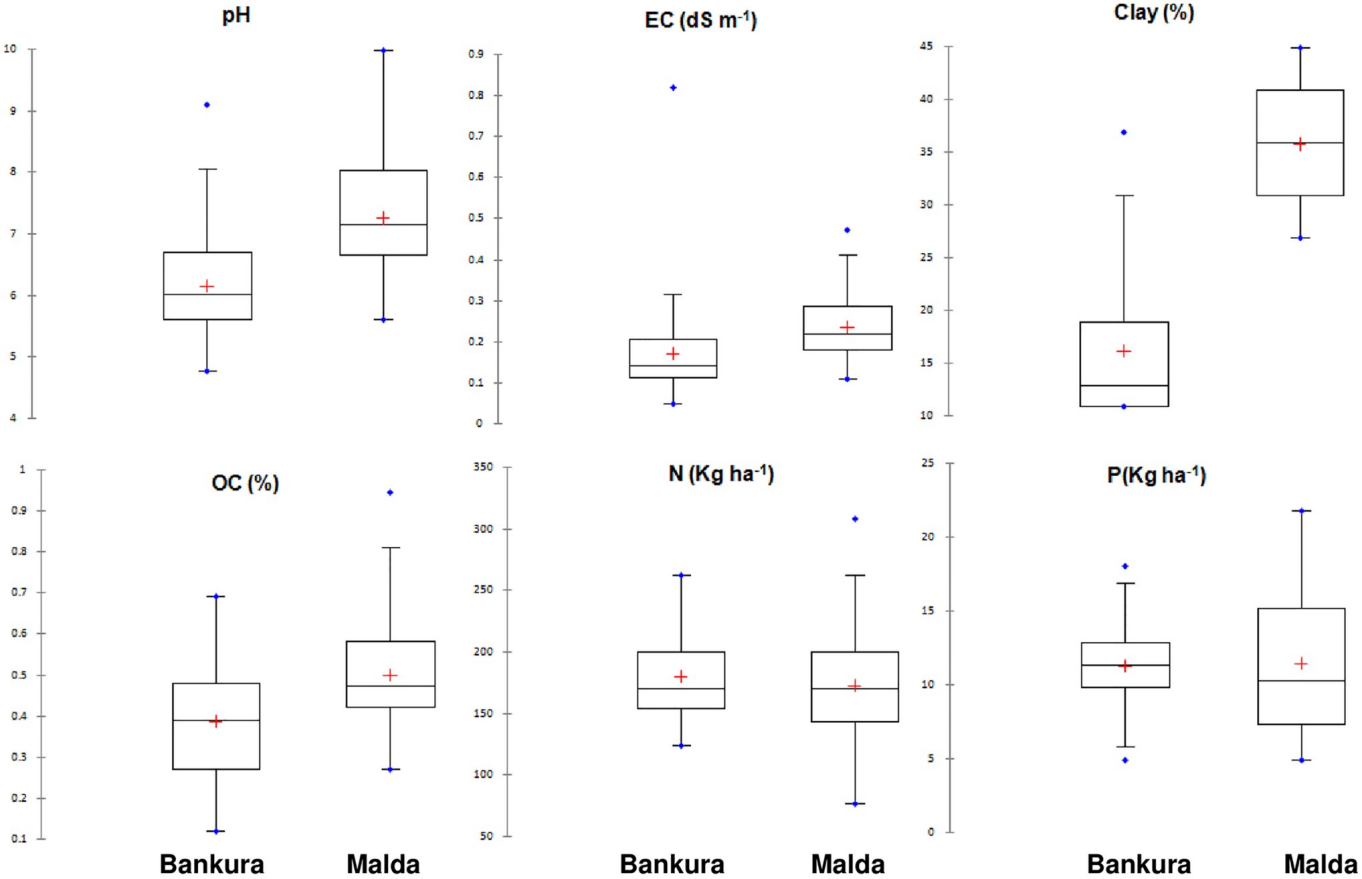
Box-and-whisker plots showing the variability of soil wet chemistry results. The mean and the median values are indicated by the cross and crossbar, respectively.

Although the overall productivity of Malda (3.79 t ha^-1^) surpassed the overall productivity of Bankura (3.41 t ha^-1^) by 11.14%, no significant yield difference was observed between them. Pooled total maize yield varied from 0.11–8.25 t ha^-1^ with Q1, Q2, Q3, and Q4 ranging from 0.11–1.86, 1.86–4.0, 4.0–4.81, and 4.81–8.25 t ha^-1^, respectively. Considerable variation in soil properties was apparent between districts. Malda had finer-textured soils with higher OC (21% higher), EC (61.54% higher), and pH (18.33%) (Fig 1). On the contrary, both the districts had similar median available N (160 kg ha^-1^) with larger interquartile range (37.5% larger) was found in Malda. A similar trend was obtained for available P.

The statistical moments of all measured soil variables for pooled data are shown in Table 3. Except for available S, considerable variability was observed for pH (4.8–10), EC (0.0–0.8dS m^-1^), available N (77–308 kg ha^-1^), available P (4.9–21.7 kg ha^-1^), available K (24.0–432.9 kg ha^-1^), clay (10.8–44.8%), sand (17.2–83.2%), and silt (4–42%). Organic carbon was significantly correlated with all parameters except available N, P, and S.

**Table 3:** Descriptive statistics of soil wet chemistry variables.

Variable	Min	Max	Mean	Std. deviation	Correlation matrix									
					pH	EC (dS m^-1^)	OC (%)	Available N (kg ha^-1^)	Available P (kg ha^-1^)	Available K (kg ha^-1^)	Available S (mg kg^-1^)	Clay (%)	Sand (%)	Silt (%)
**pH**	4.8	10.0	6.7	1.0	-									
**EC (dS m**^**-1**^**)**	0.0	0.8	0.2	0.1	S^a^	-								
**OC (%)**	0.1	0.9	0.4	0.1	S	S	-							
**Available N (kg ha**^**-1**^**)**	77.0	308.0	175.9	36.9	NS	NS^a^	NS	-						
**Available P (kg ha**^**-1**^**)**	4.9	21.7	11.3	3.9	S	NS	NS	NS	-					
**Available K (kg ha**^**-1**^**)**	24.0	432.9	134.8	90.4	S	S	S	S	S	-				
**Available S (mg kg**^**-1**^**)**	0.2	0.9	0.3	0.1	S	S	NS	NS	NS	NS	-			
**Clay (%)**	10.8	44.8	25.9	11.7	S	S	S	S	S	S	S	-		
**Sand (%)**	17.2	83.2	54.6	24.2	S	S	S	S	S	S	S	S	-	
**Silt (%)**	4.0	42.0	19.4	12.7	S	S	S	S	S	S	S	S	S	-

^a^ S and NS are significantly and non-significantly different from 0 with α = 0.05, respectively.

### 3.2 Use of principal components as proxy for soil chemical parameters and spectra

**Fig 2:**
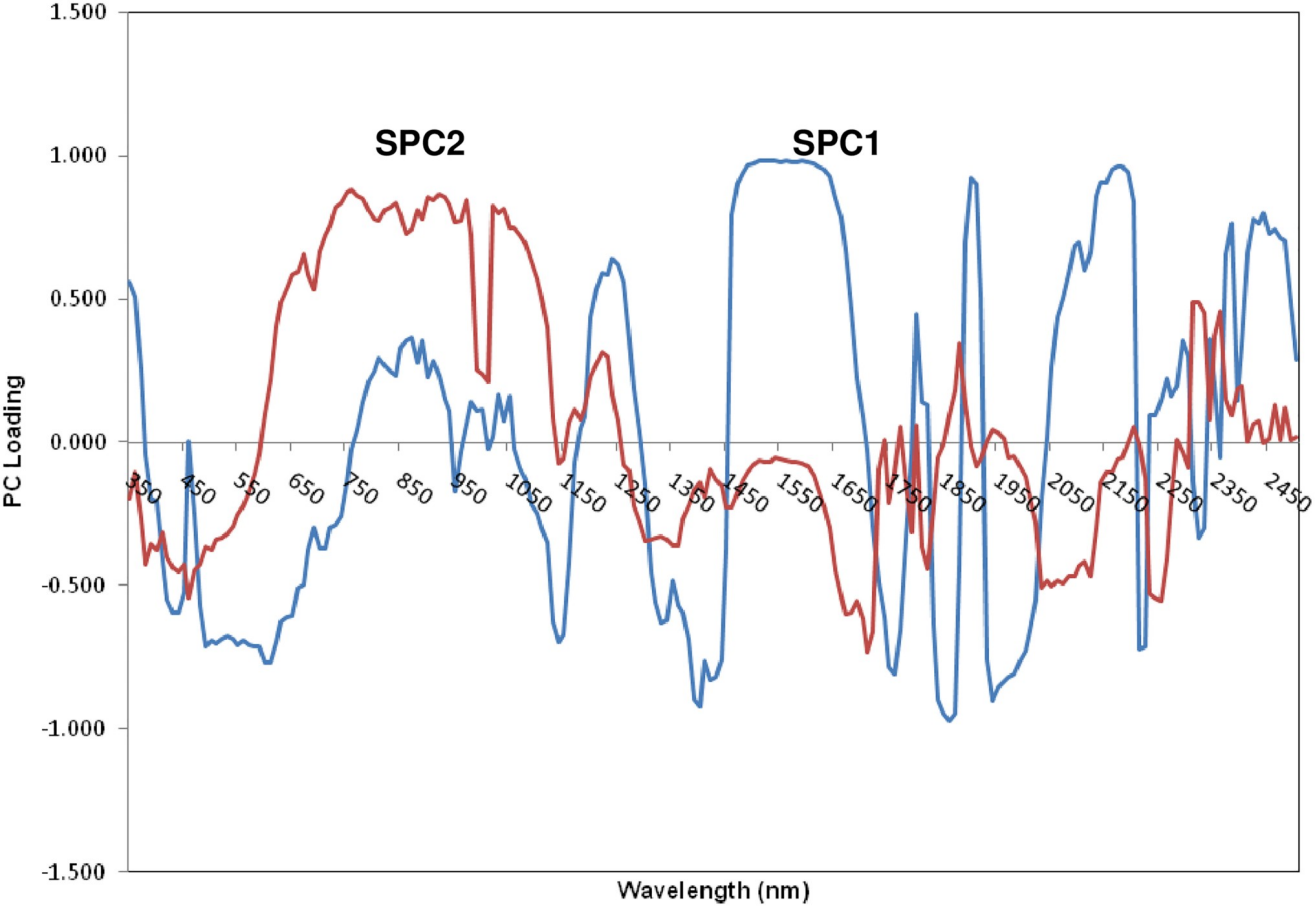
Plot showing the loadings of spectral principal component 1 (SPC1) and spectral principal component 2 (SPC2).

Principal component loadings indicated the correlation among spectral wavelengths and soil properties (Fig 2). Negative peaks in the SPC1 loadings specified the analyte of interest, and positive peaks identified interfering components [45]. The SPC1 loading weights exhibited pronounced negative contributions for wavebands between ~450–750 nm, 1050–1150 nm, 1250–1450 nm, 1700–1750 nm, 1900–2050 nm, and 2200–2240 nm, possibly arising from goethite (electronic transition), aromatics (3υ_1_) [where, υ_i_ = fundamental mode], clay minerals (kaolin doublet; 2υ_1a_ and 2υ_1b_), alkyl asymmetric–symmetric doublet (2υ_1_), carboxylic acids (3υ_1_), smectite (υ_1_+δ_a_ or υ_1_+δ_b_) or illite (υ_1_+δ), respectively [46]. Conversely, SPC2 loading weights indicated a negative contribution for ~1250–1850 nm and 1950–2150 nm regions to varying magnitudes, arising from aromatics (3υ_1_) and amides (3υ_1_). The shoulder at 2137 nm indicated polysaccharides like cellulose etc., which are part of the hard to decompose organic C.

### 3.3 Classification and regression tree for selecting influential variables

**Fig 3:**
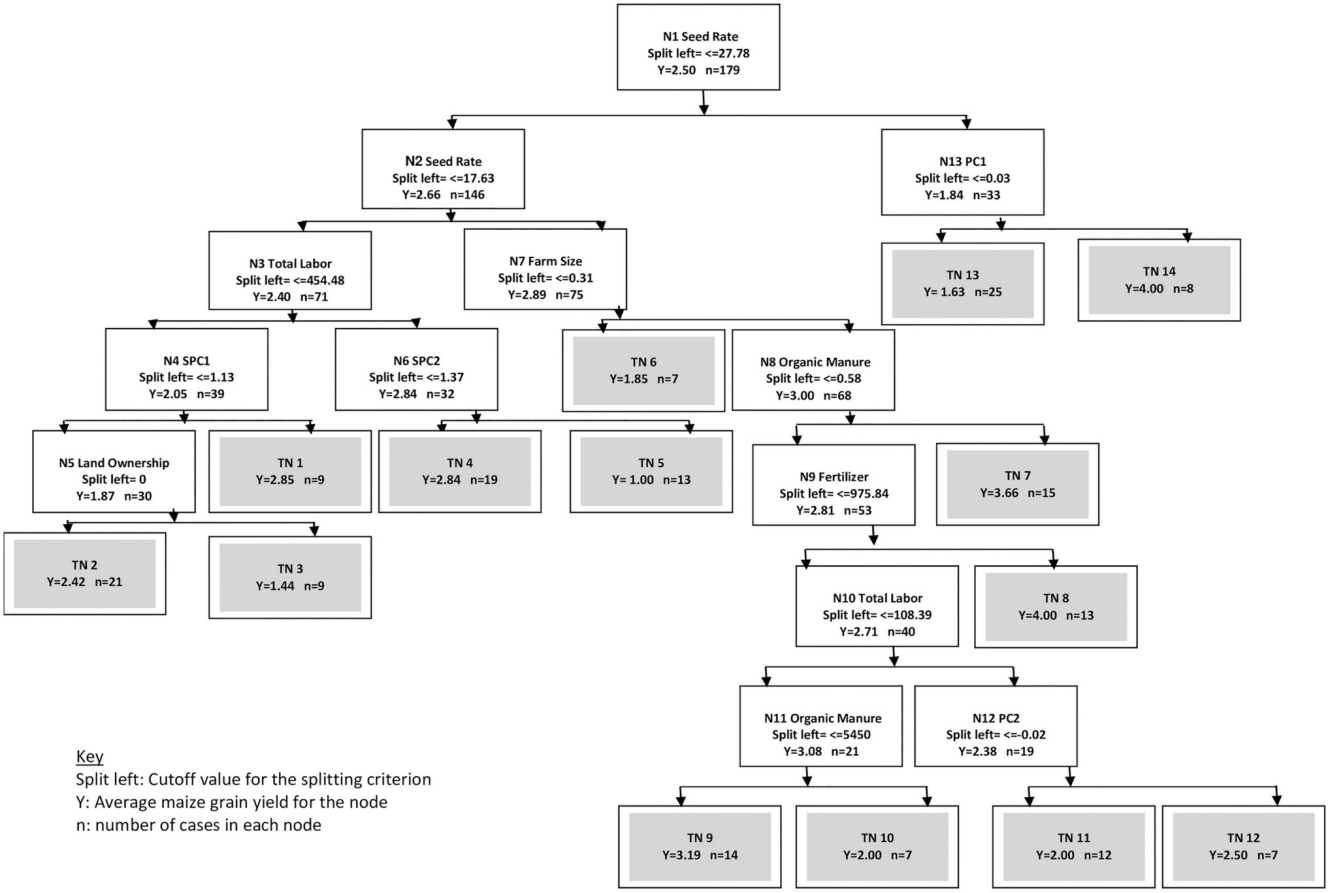
C&RT model overview for explaining maize yield variation. Splitting nodes, terminal nodes are denoted by N and TN, respectively. For more details on C&RT interpretation, see [14].

To help categorize the maize yield dataset, we explained the variability arising from the interactions among socio-economic, crop management, and biophysical variables. The whole dataset was used for C&RT analysis (n = 179), with total maize grain yield as the target variable (Fig 3). The tree had 14 terminal nodes (TN) where seed rate was the primary splitting node. Average maize yield was 2.66 t ha^-1^ at a seeding rate of <27.78 kg ha^-1^. Average yield decreased (1.84 t ha^-1^) at a higher seeding rate perhaps due to differences in the methods of sowing, leading to differences in competition for resources among the plants. At splitting node 2, as expected, lower seeding rate (<17.63 kg ha^-1^) resulted in lower average yield (2.40 t ha^-1^), and comparatively higher yield was obtained with >17.63 kg ha^-1^ seeding rate, which was further separated by farm size (splitting node 7). A combination of farm size above 0.31 ha with an application of organic manure above 0.58 t ha^-1^ showed a synergistic effect in maize yield (TN 7, average yield = 3.66 t ha^-1^). This trend can be attributed to large farmers who applied both organic and inorganic nutrient sources in sufficient amounts. The majority of cases had low (<0.58 t ha^-1^) organic manure use (n = 53). There are several constraints to sourcing organic manure in this region, such as farm size, inconvenience of organic techniques, unavailability of biomass, higher production risk, lack of training of organic practices etc. [47]. The latter group was further split by inorganic fertilizer use (sum of urea, SSP and MOP), where fertilizer applied at rates above 975.84 kg ha^-1^ produced an average yield of 4 t ha^-1^ (TN 8). Average yield (2.71 t ha^-1^) declined with lower rates of fertilizer, which represented the majority of cases (n = 40). This node was, in turn, again divided by total labor (node 10). All four soil variables (PC1, PC2, SPC1, SPC2) appeared as splitting criteria at different hierarchy levels, indicating that these were the dominant variables influencing yields.

**Fig 4:**
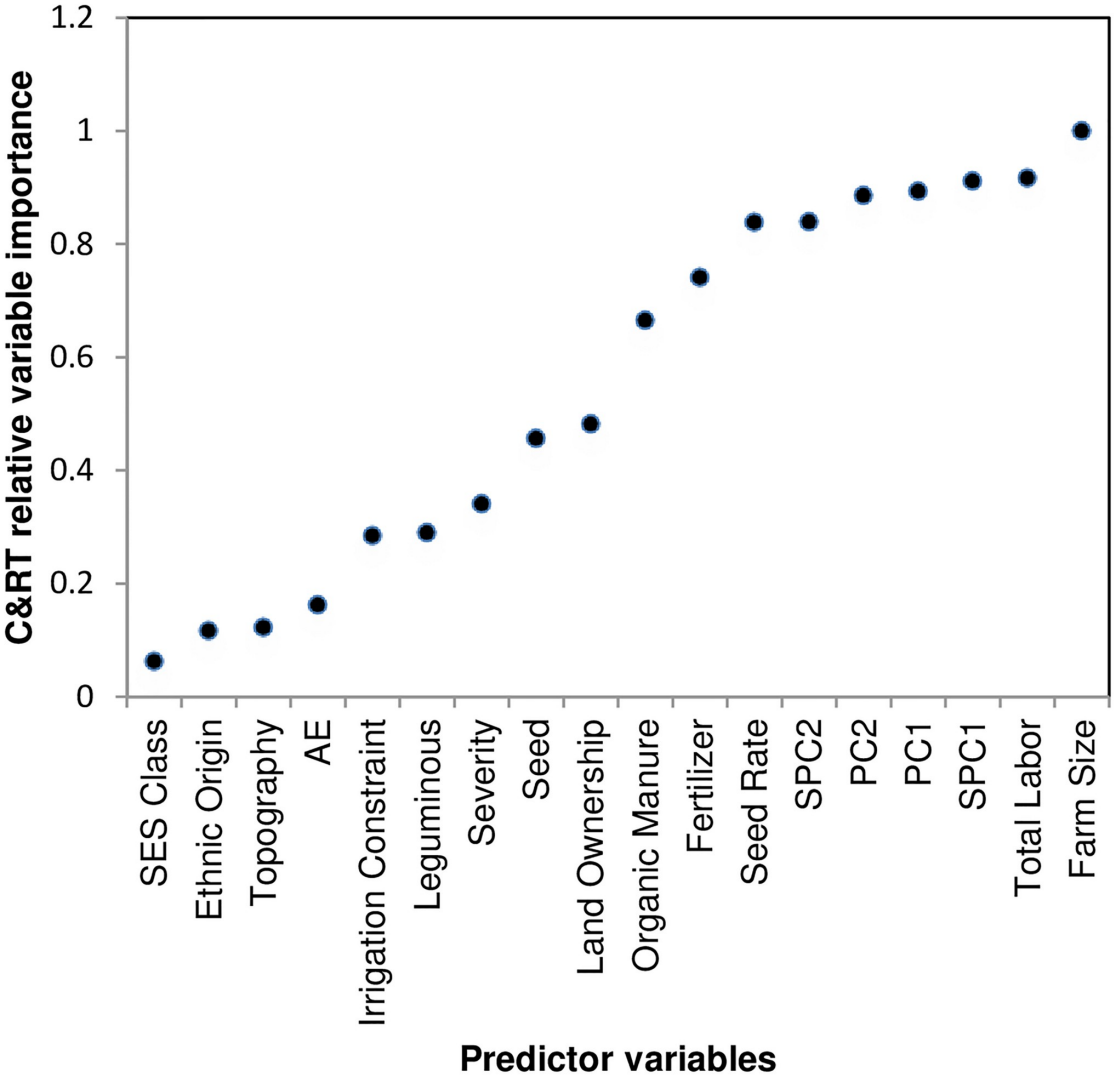
C&RT relative variable importance plot for explaining maize yield variation.

The relative variable importance plot identified the key biophysical and management factors (Fig 4). Only those variables which have relative importance > 0.05 were retained for simplicity. Farm size and total labor were the two most influential variables identified, followed by soil variables. The other important variables which have predictor importance >0.6 were seed rate, fertilizer, and organic manure. All these three factors represent the management intensity of maize cultivation.

The C&RT illustrates the complexity of the data structure and the need to categorize the yield variability arising from multiple interactions between different variables. Only the first nine C&RT predictors (>0.5 relative importance) were retained and used in subsequent classifications. Simplification of a complex dataset having large genotype-by-environment interaction [48] [74] or reduced number of inputs variables for modeling crop yield [49,50] have been common in the available literature, and the same has been applied to our dataset.

### 3.4 Classification

The RF perfectly classified the yield data with 0% misclassification both on the whole dataset and the 75% training set (n = 135) (Table 4). Conversely, the 25% test set (n = 44) exhibited lower classification accuracy with a 38% misclassification rate, classifying all but 17 samples by yield classes. Classification by SVM almost resembled the RF classification when the full dataset was used, producing 0.5% misclassification. On the contrary, the 25% test set (n = 44) exhibited lower classification accuracy than its RF counterpart, exhibiting a 47% misclassification rate. The SVM misclassification rate for the training set (n = 135) was much worse than the RF training set misclassification rate. Overall, the ANN-MLP classification model had the best performance, producing the smallest misclassification rate on the test set (25%). The overall performance of the algorithms for predicting maize yield classes can be ranked as: ANN> RF> SVM.

**Table 4:** Confusion matrix showing classification of maize yield using the random forest (RF) and support vector machine (SVM), and artificial neural network (ANN) classifications. The weighted kappa coefficients are also given (n = 179).

**RF**	**Using whole dataset (*κ* = 1)**				**75% training set (*κ* = 1)**				**25% test set (*κ* = 0.63)**			
	**Measured Q1**	**Measured Q2**	**Measured Q3**	**Measured Q4**	**Measured Q1**	**Measured Q2**	**Measured Q3**	**Measured Q4**	**Measured Q1**	**Measured Q2**	**Measured Q3**	**Measured Q4**
**Predicted Q1**	**46**	**0**	**0**	**0**	**35**	**0**	**0**	**0**	**8**	**1**	**2**	**0**
**Predicted Q2**	**0**	**44**	**0**	**0**	**0**	**33**	**0**	**0**	**1**	**8**	**2**	**4**
**Predicted Q3**	**0**	**0**	**44**	**0**	**0**	**0**	**34**	**0**	**1**	**1**	**4**	**1**
**Predicted Q4**	**0**	**0**	**0**	**45**	**0**	**0**	**0**	**33**	**1**	**1**	**2**	**7**
	**Overall misclassification**			**0%**	**Overall misclassification**			**0%**	**Overall misclassification**			**38%**
**SVM**	**Using whole dataset (*κ* = 0.98)**				**75% training set (*κ* = 0.59)**				**25% test set (*κ* = 0.54)**			
	**Measured Q1**	**Measured Q2**	**Measured Q3**	**Measured Q4**	**Measured Q1**	**Measured Q2**	**Measured Q3**	**Measured Q4**	**Measured Q1**	**Measured Q2**	**Measured Q3**	**Measured Q4**
**Predicted Q1**	**46**	**0**	**0**	**0**	**18**	**3**	**1**	**2**	**6**	**0**	**0**	**0**
**Predicted Q2**	**0**	**44**	**1**	**0**	**3**	**18**	**4**	**2**	**1**	**2**	**1**	**2**
**Predicted Q3**	**0**	**0**	**43**	**0**	**4**	**7**	**17**	**8**	**1**	**2**	**8**	**1**
**Predicted Q4**	**0**	**0**	**0**	**45**	**8**	**6**	**11**	**23**	**5**	**6**	**2**	**7**
	**Overall misclassification**			**0.50%**	**Overall misclassification**			**43%**	**Overall misclassification**			**47%**
**ANN**	**Using whole dataset (*κ* = 1)**				**75% training set (*κ* = 1)**				**25% test set (*κ* = 0.76)**			
	**Measured Q1**	**Measured Q2**	**Measured Q3**	**Measured Q4**	**Measured Q1**	**Measured Q2**	**Measured Q3**	**Measured Q4**	**Measured Q1**	**Measured Q2**	**Measured Q3**	**Measured Q4**
**Predicted Q1**	**46**	**0**	**0**	**0**	**35**	**0**	**0**	**0**	**9**	**0**	**1**	**0**
**Predicted Q2**	**0**	**44**	**0**	**0**	**0**	**33**	**0**	**0**	**1**	**9**	**0**	**1**
**Predicted Q3**	**0**	**0**	**44**	**0**	**0**	**0**	**34**	**0**	**1**	**1**	**7**	**1**
**Predicted Q4**	**0**	**0**	**0**	**45**	**0**	**0**	**0**	**33**	**0**	**1**	**2**	**10**
	**Overall misclassification**			**0%**	**Overall misclassification**			**0%**	**Overall misclassification**			**25%**

**Fig 5:**
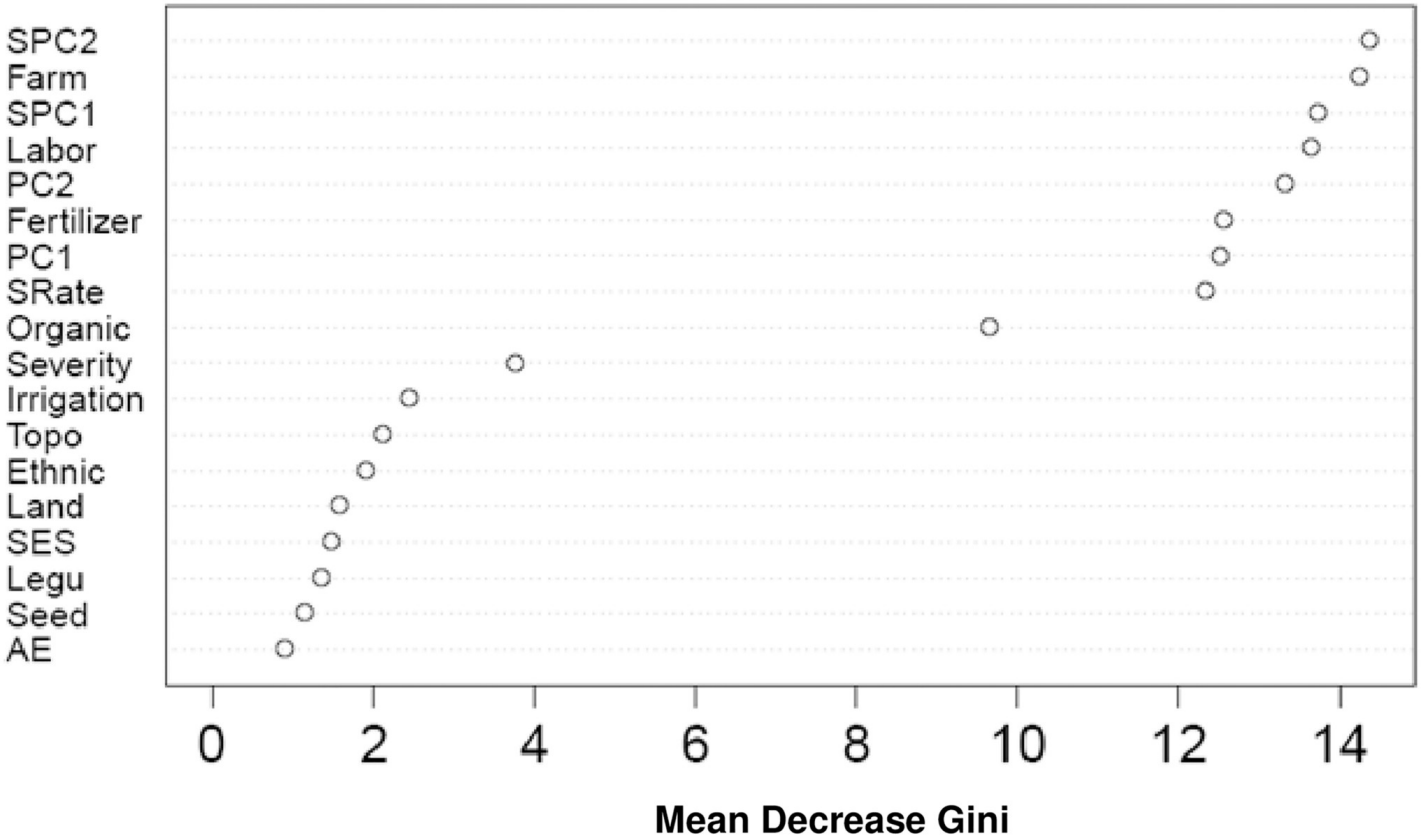
The random forest relative variable importance plot using the whole dataset based on the Gini criterion. Farm size, agro-ecological region, seed type, legume crop, socio-economic status class, land ownership, ethnic origin, topography, irrigation constraint, severity of soil problem, organic manure, seed rate, and total labor are denoted as Farm, AE, Seed, Legu, SES, land, Ethnic, Topo, Irrigation, Severity, Organic, SRate, and Labor, respectively.

**Fig 6:**
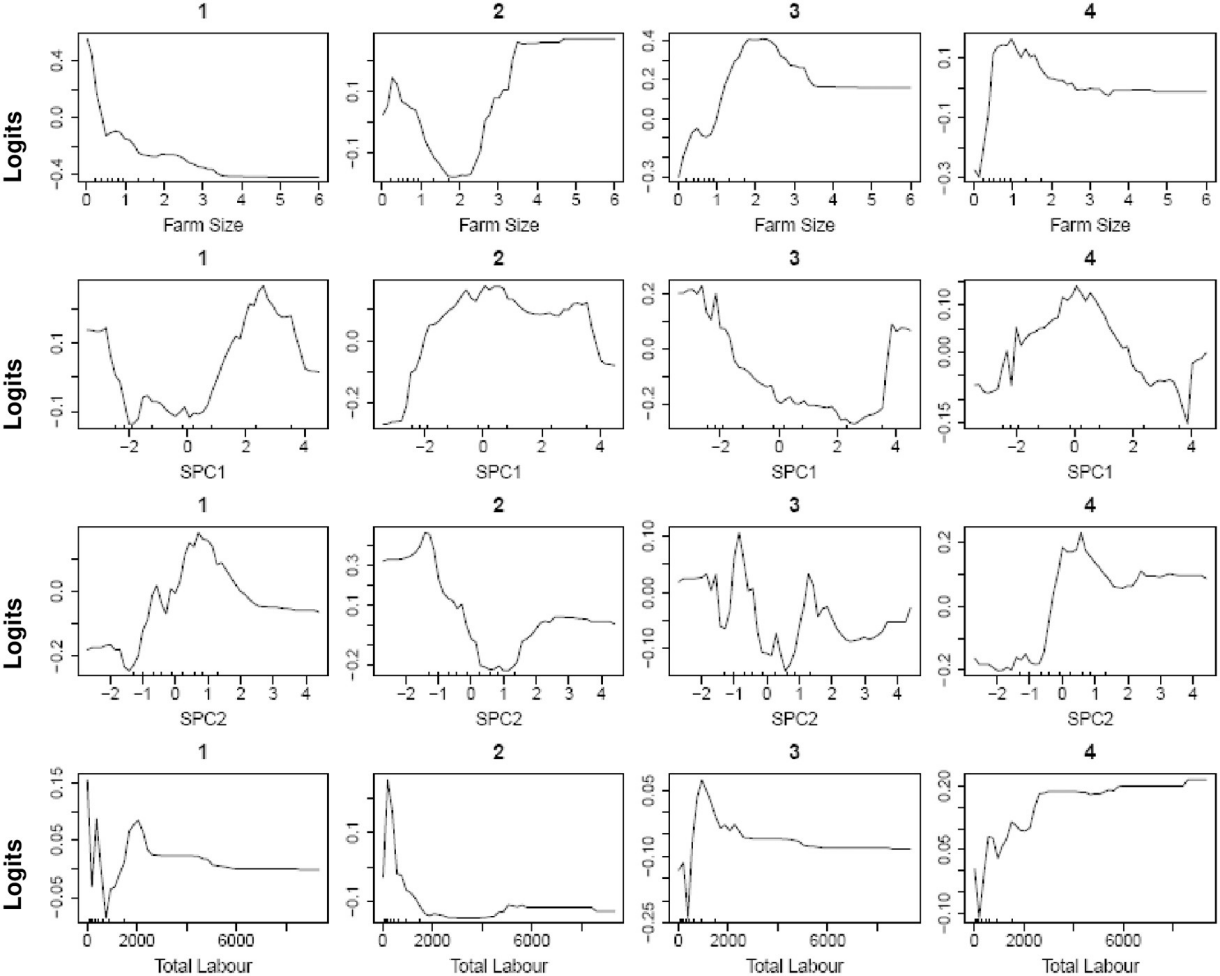
Partial dependence plot on the leading four influential variables (farm size, SPC1, SPC2, and total labor), as identified in classification and regression tree relative variable importance plot (cf. Fig 6). At the top, 1,2,3,4 (individual class) represents the 1^st^ quartile (Q_1_), 2^nd^ quartile (Q_2_), 3^rd^ Quartile (Q_3_), and 4^th^ quartile (Q_4_) of total maize yield, respectively.

**Fig 7:**
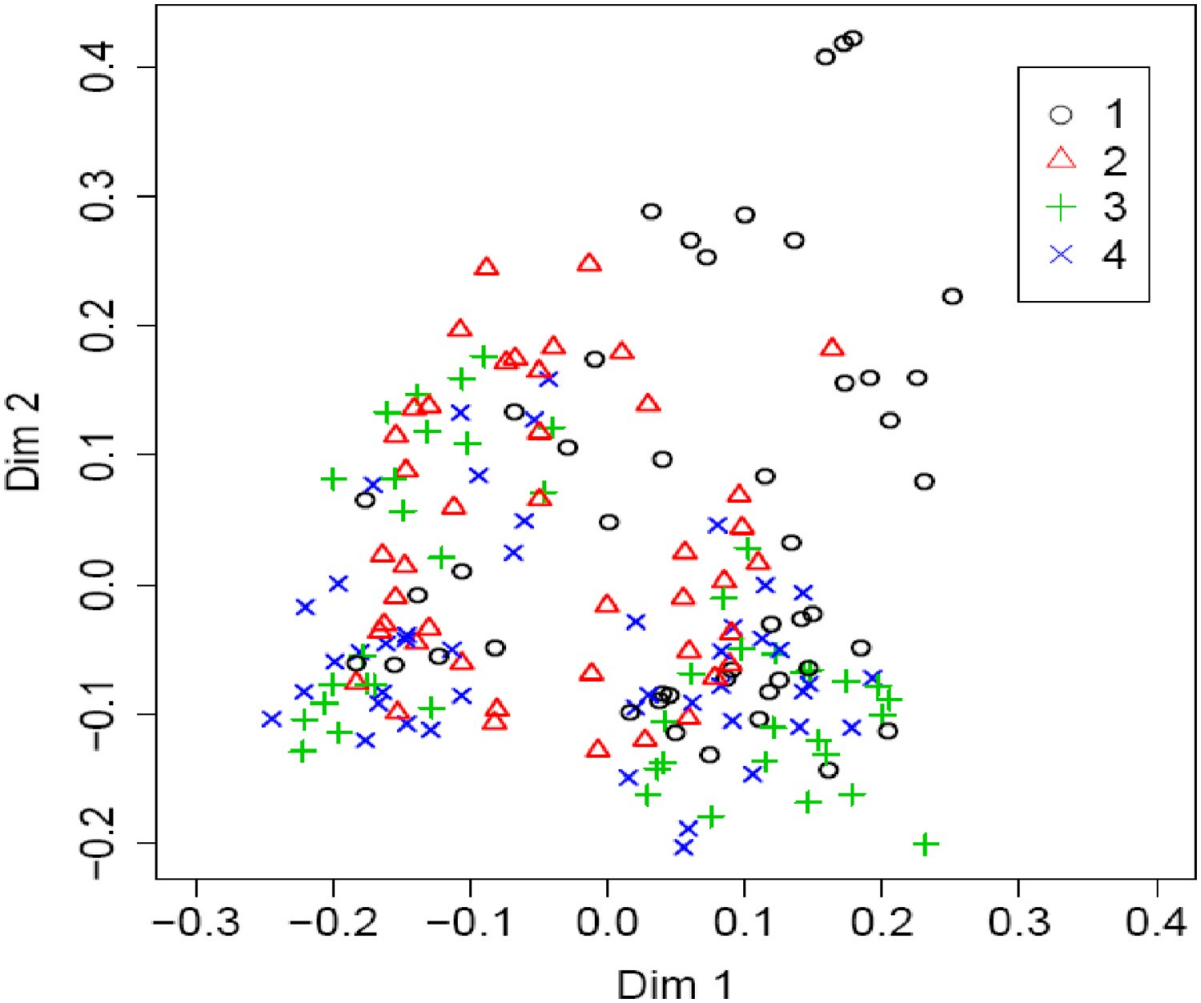
Proximity plot for the random forest classifier using the whole dataset (n = 179). Four different categories: 1,2,3,4 (individual class) represent 1^st^ quartile (Q_1_), 2^nd^ quartile (Q_2_), 3^rd^ Quartile (Q_3_), and 4^th^ quartile (Q_4_) of total maize yield, respectively. The proximity matrix is represented by two dimensions (Dim 1 and Dim 2) using multidimensional scaling.

Using the whole dataset, the RF relative variable importance analysis based on the Gini criterion exhibited an interesting trend. The leading influential variables were all the numeric variables that complemented the C&RT important predictors (Fig 5), although with a slightly different ranking. Furthermore, Fig 6 shows the partial dependence plot of the four leading influential variables (farm size, SPC1, SPC2, and total labor), as identified in Fig 5. Since the response variable (total maize yield) had four categories, each variable had four partial dependence functions, one for each class. For example, for variable farm size, it was revealed that for logits (i.e., the log of fraction of votes) of having class 1 (Q1, the first quartile of the total maize yield), total yield decreased sharply when the farm size increased from a low value. The rate of decrease in the logit slowed down when the farm size was larger. Note that the hash marks at the bottom of the plot indicated the deciles of the variable (e.g. farm size). Therefore, during interpretation, more attention was given to the dense area of the hash marks instead of the sparse area (e.g. when the farm size was greater than 2). Farm size and maize productivity demonstrated a positive relationship at different scales of farm size. This was rather interesting, indicating the differential magnitude of such association for both smallholders and relatively larger farmers. In the first plot, the initially high probability of being in class 1 (low production) may reflect the inability of smallholders to apply sufficient levels of farm inputs. This trend, however, slowed down for greater farm sizes due to the diminishing returns to production inputs [51]. While visualizing the SPC1 effect, a decreasing score below 2 (i.e. increasing impacts of soil organic matter and clay) was accompanied by a consistently increasing probability of being in class 3 (higher yield). With a decreasing SPC2 score, a sharp increase in probability was observed in classes 2 and 3 for most cases. We produced the RF proximity plot using the whole dataset to observe the clustering structure among the samples and to identify the outliers in the data, we produced the RF proximity plot using the whole dataset, which gave an indication of the observations that were effectively close together, as determined by the random forest classifier (Fig 7). Note that a proximity plot is based on similarities between cases, i.e. the number of times that cases were placed in the same terminal nodes [52]. However, in our case, a big overlap was observed between classes 1 and 2, with three outliers (on the upper right) in class 1, although intraclass variability was evident from the sparse nature of cases. Further, both class 3 and class 4 seemed to have two subclasses.

**Fig 8:**
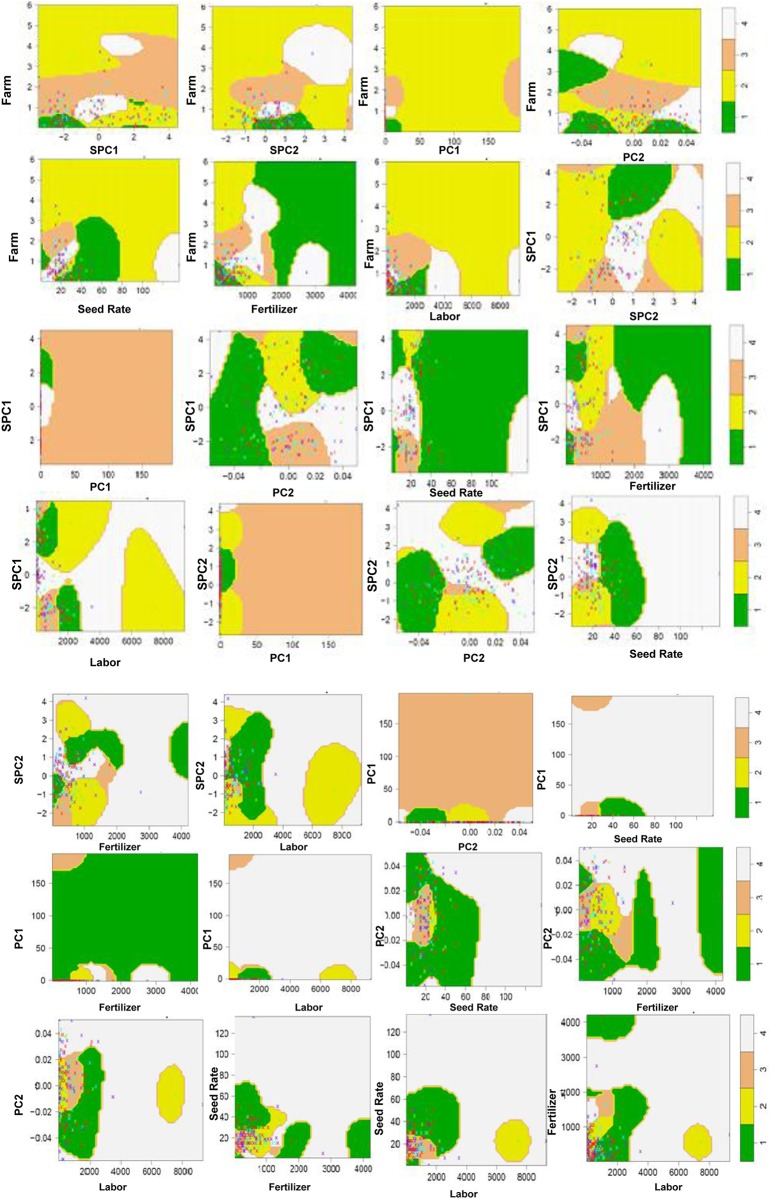
Complex interactions through nonlinear support vector machine (using the Gaussian kernel) boundary for the whole data with the leading eight influential variables, as identified in classification and regression tree relative variable importance plot (cf. Fig 6). Four different categories: 1,2,3,4 (individual class) represent 1^st^ quartile (Q_1_), 2^nd^ quartile (Q_2_), 3^rd^ Quartile (Q_3_), and 4^th^ quartile (Q_4_) of total maize yield, respectively.

Fig 8 illustrates complex interactions through nonlinear SVM (using the Gaussian kernel) boundary for the leading eight influential variables, as identified in Fig 5. We used only the bivariate model since it is very difficult to visualize the boundary for a model with more than two variables. The nonlinearity and complex interaction for the SVM boundary in the SVM pairwise plot were apparent. For example, in the first plot (farm size vs. SPC1), class 2 dominated the upper (large farm size) and lower right corner (small farm size and large SPC1 value). Class 3 spanned the range of SPiC1 with median values for farm size. Class 4 had two clusters, both having SPC1 values around zero. The class 1 area appeared when farm size was small and the majority area was at the lower-left corner (small farm size and small SPC1 value). While visualizing the interaction between SPC2 with farm size, it was obvious that although class 2 spanned the range of farm sizes, the coverage under class 2 was more pronounced when farm size exceeded 2 ha (large farm size). One noticeable pattern was the clustering tendency of class 1 (lower yield) around small farm size in the first seven plots, except the farm vs. SPC2 plot. Excessive use of fertilizer lowered the yield irrespective of farm size (farm vs. fertilizer). A synergistic relationship between farm size and total labor for increasing yield was observed from the farm vs. labor plot. A visual inspection of SPC1 vs. SPC2, PC2, seed rate, fertilizer, and labor revealed a subtle trend of clustering class 3 and 4 (higher yields) when SPC1 score tended below 0 (higher organic matter or clay). A synergistic effect between low SPC1 score and high seed rate was evident (a cluster of class 4 at the lower right corner of SPC1 vs. seed rate). Moreover, fields with the high impact of organic matter or clay produced less (class 1) after the fertilizer application reached a threshold. Seeding rate was positively related to fertilizer dose and labor, while fertilizer dose was positively correlated with total labor. Among other positive interactions, PC1 vs. PC2, PC1 vs. labor, and PC1 vs. seed rate were important, as interpreted from the distribution of class 4. In the PC1 vs. fertilizer plot, a pronounced presence of class 1 realistically revealed the negative effect of over-fertilization on a fertile field. Summarily, we suggested that the interpretation of causal relationships needed a cautious approach, because many biophysical and management variables seemed to be highly correlated with each other.

### 3.5 Yield prediction

**Fig 9:**
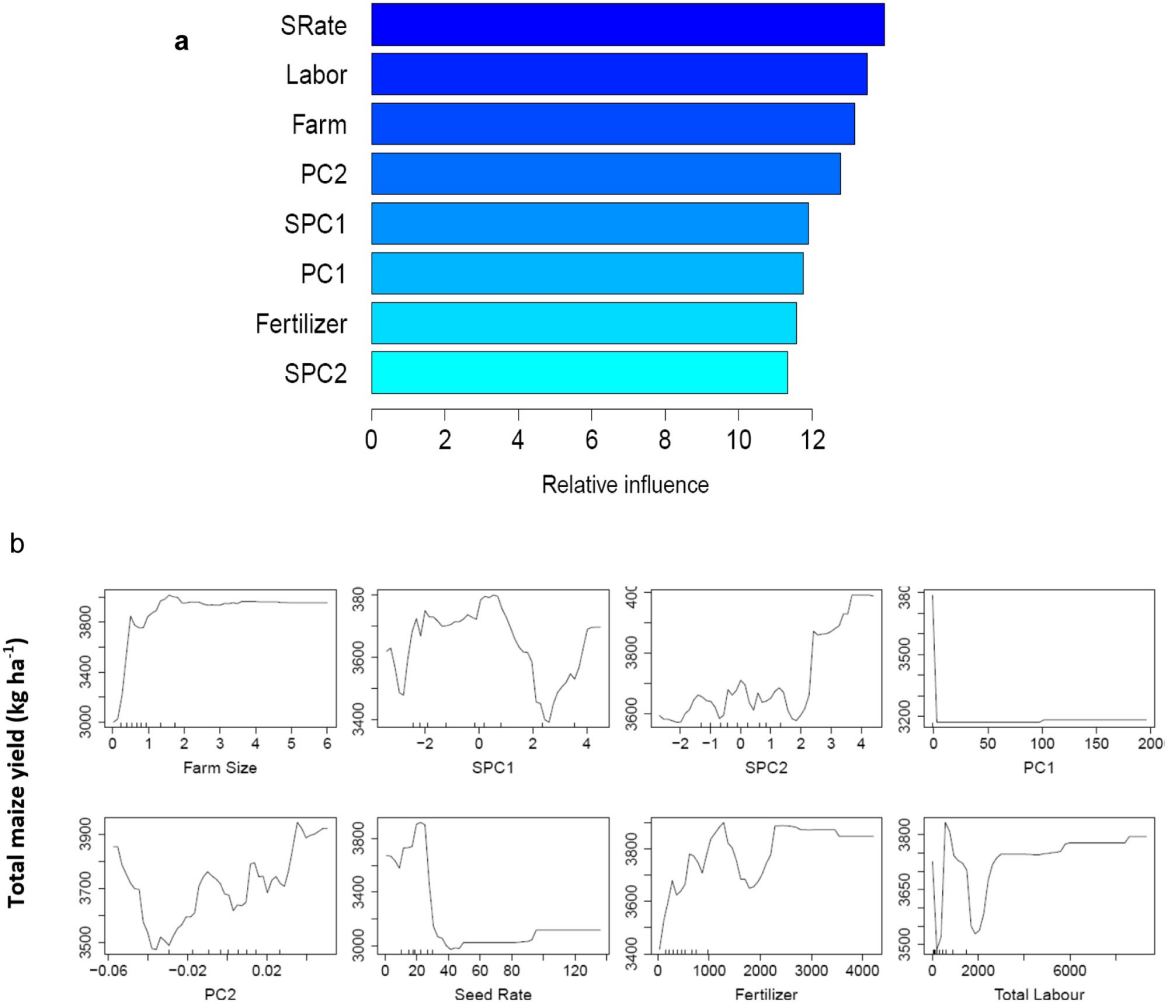
Plots showing a) RF variable importance plot where explanatory variables are arranged according to their relative contribution to the overall prediction process. Seed rate, farm size, and total labor are denoted as SRate, Farm, and Labor, respectively and b) partial dependence plots on the eight influential variables (farm size, SPC1, SPC2, PC1, PC2, seed rate, fertilizer, and total labor) used in random forest regression. The y-axes in all the plots represent total maize yield (kg ha^-1^).

The random forest regression was sufficiently able to capture the intricacy in the non-linear data structure and to predict the total maize yield, indicated by an R^2^ value of 0.94 (RMSE = 846 kg ha^-1^). The RF regression variable importance plot (Fig 9a) exhibits explanatory variables, arranged according to their relative contribution to the overall prediction process. Subsequently, the partial dependence plots of all the eight variables provided a more straightforward interpretation of the relative influence of different biophysical attributes and land management factors on total maize yield (Fig 9b). Total yield increased sharply when the farm size increased from a low value. Subsequently, the rate of increase slowed down and reached a plateau when the farm size was large. Yield increased rapidly only when the seeding rate and total labor increased from a low value. In general, a positive contribution of soil available P on total yield can be inferred when PC2 score was positive.

## 4. Discussion

Yield variability of maize among different villages within a block was inherently large, likely due to difference in sowing dates [53], growing environments [54], and choices of cultivar [55]. The higher inter-quartile range of soil properties for Malda could be largely explained by higher variation in nutrient management intensity owing to greater socio-economic variability between sub-locations and inherent variation in soil properties. Apart from other factors, soil texture explained OC variability because of the greater capacity of soil richer in clay and silt for physicochemical carbon stabilization in soils richer in clay and silt [56].

Spectral analysis (in terms of SPC1 and SPC 2) was sufficiently sensitive to capture spectral features of soil OC and clay minerals. Assigning precise wavebands for individual soil parameters was difficult due to the complex nature of soil matrix. Consequently, determining the relationship between the size of the PC score and the loading was not straightforward. For simplicity, we used negative spectral scores for each SPC1 and SPC2 as comprehensive indicators of both soil OC and clay content. In contrast, PC1 had significantly larger positive loadings on clay (0.972) and OC (0.543) while PC2 had significantly larger positive loading on available P (0.482) than rest of the variables with minor positive and negative loadings. The larger the absolute value of loading weight, the greater the contribution of the corresponding input variable to the output. Thus, clay and OC were the most influential variables in PC1 while available P was most influential in PC2. Large positive values of PC1 represented large values of clay and OC, while large positive values of PC2 were associated with high soil available P. To aid interpretation, we used PC1 to denote the combined effect of clay and OC while PC2 denoted available soil P.

In the C&RT analysis, the appearance of seeding rate as the primary splitting node stems from the fact that most of the farmers in Malda sow maize seeds with specific row arrangements (line sowing method) while the farmers of Bankura prefer broadcasting with a higher seeding rate. Data suggested optimum seeding rates in the range of 17.63–27.78 kg ha^-1^ (the wide range might be attributed to variations in biophysical conditions of farms), which is close to the recommendations of the state department of agriculture. It is well known that optimum plant stand is key to achieving resource use efficiency and higher productivity in maize, and this is critical in situations where farm resources are scarce and optimum nutrient management is not assured [57]. Nutrient management in relation to soil fertility variation is perhaps the most important factor influencing maize yield gap [9, 58, 59], and fertilizer is probably the easiest but the costliest option to offset soil fertility constraints for maize productivity [16, 60]. However, its return to maize yield is a complex phenomenon that goes beyond cost-benefit rationale [61, 62].

The variable ‘agro-ecological region’, represented by two districts, was not selected by C&RT as an explanatory variable, suggesting that site effects were explained instead by the biophysical and management variables. Notably, it was observed that seeding rate, organic manure, and total labour showed more than one threshold value that reappeared as splitting criteria, signifying their multi-modal distribution in the dataset. These variables did not have a monotonous relationship with maize yield and had optimal quantitative ranges associated with higher maize yields (in combination with ranges of other variables). This was expected since maize yield variability, like that of many other crops, is governed by complex interactions of climatic, socio-economic, and crop management practices [39, 63, 64].

According to Tittonell et al. [65], soil fertility and fertilizer use can be used as reliable proxy measurements to explain yield variability This conclusion was corroborated by the C&RT variable importance plot (Fig 4).

Yield variability was also attributable to differences in farm size and productivity. Efficiency of farm size increases with the number of family members of working age and with the household’s working capital or resource endowment [66]. Farm size is widely believed to be related to the adoption of new technologies and to crop productivity [67, 68, 69, 70]. The nature of such relationships is subject to debate and depends on the level of technology being employed in farming. The efficiency of input management is reported to have a positive relationship with farm size when crop management is technology-intensive [71, 72]. Moreover, resource-rich, large farmers have better access to credit [67, 73] and are believed to invest more in maize production, especially in external sources of plant nutrients [58] [55]. Literature also suggests that large farmers are more likely than smallholders to adopt improved technologies [70, 74], and thus more likely to achieve higher yield. Since maize is grown as a cash crop (by selling to the animal feed industry), it requires higher management intensity that can mostly be maintained by resource-rich farmers when input support from public extension agencies is either absent or insignificant. The family remains the main source of farm labor in small farms. Notably, efficiency of small family farms depends on the extent of family labor use, which is not available to the increasingly divided nuclear families. This is critically challenging to policymakers, since the majority of farm households studied operated in farms below one hectare in size. Among other influential factors observed in the C&RT (Fig 4), organic manure provides both crop nutrient needs and improves soil health, both of which are necessary for sustaining long-term productivity [75, 76]. Management factors are also influenced by climatic and biophysical conditions under different socio-economic settings of farmers.

A misclassification rate between 47 and 0% is large but realistic, based on the complex interactions among several biophysical, management, and socio-economic factors affecting maize yield. Such interactions are common in smallholder systems [63,77], which are often non-linear, have differential trends at different magnitudes, and affected by outliers. The rate of misclassification was also found to be close to other studies [78]. More samples in the training set could have improved the probability of a better classification. Furthermore, the intricate initial parameterization of ANN needs due consideration.

In the RF proximity plot (Fig 7), farms having low yields showed intra-class variability in terms of differing farm sizes, resource-endowments, soil fertility gradients, management intensities, and interactions among them. Larger yield classes (mostly large holders) are likely grouped by agro-ecological zones or growing seasons, but their effects are largely masked by highly variable management intensity among smallholders.

Although RF regression was able to satisfactorily predict maize yield, we refrained from over-interpreting the model prediction accuracy. Our main objective was to test the capability of a new methodological framework to help explain different factors and their interactions that affect maize yield. Our objective was not to develop a laboratory-grade predictive model. Moreover, Jame and Cutforth [26] argued that more than 10 years of continuous data are often required to confidently predict crop growth in any empirical model. Seasonal and cross-seasonal validations were beyond the scope of this study, due to data insufficiency and requirements for future experiments to draw stronger conclusions. Additionally, retaining a part of the dataset for cross-validation to prevent overfitting is not a desirable characteristic for an empirical model building tool [21].

In the RF partial dependence plots (Fig 9b), the positive relationship between farm size and total yield may be explained from low input use of smallholder farmers. Typical explanations for lower yields on small farms are diminished returns, the presence of frictions in the land, and reduced access to credit and insurance markets [51]. Small farmers do, however, often have advantages in labor supervision because of their high reliance on family labor [79,80]. For resource-rich farmers, increased marginal costs of supervision can result in higher land to labor ratios and lead to decreased output per unit area, even though farm size is larger. Seeding rates higher than optimum increase competition among plants for resources, lead to plateaus or even decreases in maize yield [81]. Note that a sharp dip of total yield was identified at the beginning of the yield vs. labor curve in this study. This fact suggests a typical diminishing marginal return on labor, presumably due to under-employed family labor spending less productive hours on their own farm. This is common in smallholder farms when farming is not highly technology-driven. A somewhat similar trend with farm size was observed with fertilizer, where a sharp decrease in yield occurred after a certain level of fertilization was achieved, perhaps due to nutrient imbalance [82], which is common in many parts of eastern India. Since there is a subsequent rise in yield after the sharp decline, the decline might be attributed to a given geographical region where imbalanced fertilization is common among farmers.

## 5. Conclusion

The yield gap of maize in eastern India is a complex interplay of climatic variations, soil fertility gradients, differential management intensities and farmer socioeconomics. With an increasing shift to maize-based cropping systems in eastern India replacing the conventional rice-based system, understanding maize yield determinants has become critical for creating effective interventions. This study has drawn upon a host of complex interacting yield determining factors, using machine learning approaches like PSR, C&RT, RF, SVM, and ANN to identify important biophysical, socio-economic, and crop management factors for explaining maize yield. The C&RT relative variable importance plot identified farm size, total labor, soil factors, seed rate, fertilizer, and organic manure as influential factors. Among three classification approaches compared for classifying maize yield classes, ANN produced the smallest misclassification rate on the test set and outperformed RF and SVM. In the RF classification scheme, all the numeric variables appeared as the leading influential variables to classify maize yield. Moreover, the RF partial dependence plots exhibited a positive relationship between farm size and maize productivity. A nonlinear SVM boundary for the leading eight influential variables revealed complex interactions between influential factors in determining maize yield response. These algorithms may be used both in future empirical studies and in developing efficient crop simulation models for ex-ante yield estimations of field crops.

## Supporting information

S1 File (DOC)

S2 File (DOC)

## Abbreviations

ANN: artificial neural network
C&RT: classification and regression tree
PSR: penalized spline regression
RF: random forest
SVM: support vector machine
VisNIR DRS: visible near-infrared diffuse reflectance spectroscopy
