# Supplementary material for: Maize yield in smallholder agriculture system—An approach integrating socio-economic and crop management factors
Source: PLoS One. 2020 Feb 24;15(2):e0229100. doi: 10.1371/journal.pone.0229100 (PMC7039445; doi:10.1371/journal.pone.0229100)
Supplement: S1 File — (DOC) [file pone.0229100.s001.doc]

**Maize Farmer Typology Survey Questionnaire for Eastern India – 2012**

(IPNI, CIMMYT, DMR, BAU (Bihar), BAU (Jharkhand), OUAT (Odisha), BCKV (WB)

Date of Interview:__________________________ Name of Interviewer: ____________________________________

Name of Farmer:__________________________ Location: State:_______________ District: ________________ Block _________

Village:­­­­­­­­­­­­______________ Latitude:____________, Longitude:________________

Type of Farmer: Cooperator ____ Non-cooperator _____ If Cooperator, check type of plot: Farmer’s Practice ______ Experimental _______

# I. SOCIO-ECONOMIC PROFILE

# A. Farmer Profile

1. Age of farmer: __________________________________________; Educational level: ____________________________________
2. Years in farming:________________________________________; Ethnic Group (Caste)______________________________________
3. Household size: __________; No. of members working in the farm:_____________; No. of member working off-farm_______________
4. Non-farm sources of income: Daily wages________; Government job____________; Private job______________
5. Estimated annual income: Farm______________________________; Non-farm________________________________
6. Tenure status (Pls check): Landowner____________; Share Tenant __________; Leasee __________; Farm worker ________________
7. Number of Livestock: Total_______; Cow/Buffalo_______; Goat/Sheep______; Poultry/Duck_______
8. Do you own a pond: Yes_________; No__________; Is Fisheries a source of income: Yes__________; No____________;

# B. Farm Profile

1. Total size of farm (ha): ____________________________ Number of plots: ______________________
2. Cropping System:
3. Plot 1 (______ ha) Kharif __________________ Rabi____________________ Spring/Summer ______________
4. Plot 2 (______ ha) Kharif __________________ Rabi____________________ Spring/Summer ______________
5. Plot 3 (______ ha) Kharif __________________ Rabi____________________ Spring/Summer ______________
6. Plot 4 (______ ha) Kharif __________________ Rabi____________________ Spring/Summer ______________
7. Plot 5 (______ ha) Kharif __________________ Rabi____________________ Spring/Summer ______________
8. Did you start growing any new crop recently

Kharif__________________________; Rabi____________________________; Spring/Summer_____________________________

1. Distance of farm from input market (Km): _____________________ Distance of farm from output market (Km): _______________
2. Distance to metal road (Km): _____________________________________________________________________________
3. Means of transportation: __________________________________________________________________________________________
4. Source of capital for maize production (Pls. check) : a. Credit: from I) traders______; ii) bank______; iii) individuals _________

b. Savings: from farm __________________; from non-farm sources _________

1. Topography of farm (Pls. check): Flat _______________________; Sloping/Undulated ________________
2. Source of farm irrigation (Pls. check):\
3. **Irrigated** - Deep well_________; Shallow tube well ____________; Pond _______________; Others (Specify)___________________
4. **Rainfed** - Favorable ________________ Unfavorable ___________________

# C. Farm Asset Inventory

| Farm Assets | Do you own or rent the following? | If owned, indicate number of units. | If rented, indicate rental cost |
| --- | --- | --- | --- |
| Animal for draft power |  |  |  |
| Tractor |  |  |  |
| Power Tiller |  |  |  |
| Submersible Pump/Shallow Tube well |  |  |  |
| Storage |  |  |  |
| Seed drill/Planter |  |  |  |
| Dryer |  |  |  |
| Sheller |  |  |  |
| Vehicle for transport of produce |  |  |  |
| Others (Specify) |  |  |  |

# II. MANAGEMENT PRACTICES, LABOR AND MATERIAL INPUTS FOR MAIZE

**A. Labor inputs and sources of labor by farm activity** (Provide the information for a plot where maize is grown)

Size of cultivated maize parcel (Ha.) ____________________ Size of experimental plot (if applicable) : ____________

| Farm Activity | LABOR INPUTS (Indicate sources of labor and time spent) | | | | | |
| --- | --- | --- | --- | --- | --- | --- |
|  | Family | | Hired | | Exchange | |
|  | No. of days | No. of hrs/day | No. of days | Wage rate (Rs./day) | No. of days | No. of hrs/day |
| Land preparation |  |  |  |  |  |  |
| Planting  Plant spacing: ____ cm x ___ cm |  |  |  |  |  |  |
| **Fertilizer application:**  Basal |  |  |  |  |  |  |
| Top dressing |  |  |  |  |  |  |
| Earthing-up |  |  |  |  |  |  |
| **Weeding:**  Hand weeding |  |  |  |  |  |  |
| Herbicide spraying |  |  |  |  |  |  |
| **Irrigation:**  No. of irrigations |  |  |  |  |  |  |
| Insect & Disease control |  |  |  |  |  |  |
| Harvesting/Threshing |  |  |  |  |  |  |
| Drying |  |  |  |  |  |  |
| Shelling |  |  |  |  |  |  |
| Bagging/packaging |  |  |  |  |  |  |
| Storage |  |  |  |  |  |  |
| Marketing |  |  |  |  |  |  |

**B. Material inputs by farm activity** ((Provide the information for a plot where maize is grown)

Size of cultivated maize parcel (Ha.) ____________________ Size of experimental plot (if applicable): _______________

| Farm Operation |  | | |
| --- | --- | --- | --- |
|  | **Cash inputs** | | |
|  | Kind | Total Qty. applied/unit area (kg/ha) | Price/unit  (Rs/kg) |
| Planting:  Planting material/ seeds | Composite/Hybrid |  |  |
| Organic manure application |  |  |  |
| Fertilization:  i) Basal | Name of fertilizer:  a)  b)  c)  d) | a)  b)  c)  d) | a)  b)  c)  d) |
| ii) 1^st^ Top dressing | a)  b) | a)  b) | a)  b) |
| iii) 2^nd^ Top dressing | a)  b) | a)  b) | a)  b) |
| iv) Further applications (specify) |  |  |  |
| Weed control:  Herbicide spraying | Name of herbicide |  |  |
| Insect control:  1^st^ |  |  |  |
| 2^nd^ |  |  |  |
| Disease control |  |  |  |
| Drying |  |  |  |
| Shelling |  |  |  |
| Packaging |  |  |  |
| Storage |  |  |  |
| Marketing |  |  |  |

**III. OPERATIONAL COSTS IN CROPS OTHER THAN MAIZE**

| Farm Operations | Crop 1 | Crop 2 |
| --- | --- | --- |
| Seed |  |  |
| Organic Manure |  |  |
| Inorganic fertilizer |  |  |
| Weed Control |  |  |
| Plant Protection |  |  |
| Labour for all operations including post-harvest operations and marketing |  |  |

# IV. MAIZE PRODUCTION LEVELS, UTILIZATION AND DISPOSAL (FOR LAST CROP OF MAIZE)

| Maize Production | | | Maize Utilization and Disposal | | | | | |
| --- | --- | --- | --- | --- | --- | --- | --- | --- |
| Total Amount Harvested | Unit | Price/unit | Qty Sold | Qty Stored for seeds | Harvester share | Qty paid to credit | Qty for Home use | Qty fed to animals |
| Kharif |  |  |  |  |  |  |  |  |
| Rabi |  |  |  |  |  |  |  |  |
| Spring/Summer |  |  |  |  |  |  |  |  |
| Total |  |  |  |  |  |  |  |  |

# V. PROBLEMS ENCOUNTERED IN MAIZE PRODUCTION BY FARM ACTIVITIES

| Farm Activity | Problems encountered | Coping mechanisms or  Suggested solutions |
| --- | --- | --- |
|
|
| Seed |  |  |
| Planting |  |  |
| Fertilizer |  |  |
| Pesticide |  |  |
| Labor |  |  |
| Irrigation |  |  |
| Storage |  |  |
| Marketing |  |  |
| Others |  |  |

# VI. SOIL RESOURCES: CONSTRAINTS

1. Farmer-perceived soil problem(s)

| Problem^1^ | **Severity^2^** | **Estimate of maize area affected (%)** | Cause | Efforts to minimize/address the problem(ie. by farmer, local gov’t.) |
| --- | --- | --- | --- | --- |
|  |  |  |  |  |
|  |  |  |  |  |
|  |  |  |  |  |
|  |  |  |  |  |
|  |  |  |  |  |

^1^ Example: Erosion, nutrient depletion, acidification, salinization, crusting, compaction, organic C/organic matter depletion, waterlogging, biological degradation, soil pollution.

^2^ Degree or severity of problem: Light, Moderate, Strong/severe, Very strong/very severe

B. Is soil testing done on the farm? Yes ______ No ______

If yes, how often? _______________________ ; Where was it done (Govt./Private)_________________________

When was the last soil test done? ______________

What was the result (low/medium/high): pH__________; Organic Carbon______________; P_________________; K________________

# VI. WATER RESOURCE USE

1. Source(s) of irrigation:

1. Is water from source(s) enough to irrigate all crops? (Yes/No) __________
2. Area under irrigation for maize (ha): Kharif __________________ Rabi ________________ Spring/Summer __________________
3. Do you practice water conservation in your farm? (Yes/No) ______________ How? ________________________________
4. State other problems related to water use: _________________

**VII. INFORMATION ON NEW/LATEST TECHNOLOGIES**

| **S. No** | **Information** | **Source** |
| --- | --- | --- |
| 1 | Use of hybrid seeds |  |
| 2 | New planting techniques (Bed planting/Zero Tillage) |  |
| 3. | Fertilizer application (4Rs)  Right Source; Right Rate; Right time; Right Place |  |
| 4 | Weed Management practices |  |
| 5 | Plant Protection |  |
| 6 | Post Harvest Management including seed storage |  |

*End of Interview.* THANK YOU!
