# Supplementary material for: Maize yield in smallholder agriculture system—An approach integrating socio-economic and crop management factors
Source: PLoS One. 2020 Feb 24;15(2):e0229100. doi: 10.1371/journal.pone.0229100 (PMC7039445; doi:10.1371/journal.pone.0229100)
Supplement: S2 File — (DOC) [file pone.0229100.s002.doc]

# Supplementary Material

**Title: Maize (*Zea mays* L*.*) Yield in Smallholder Agricultural Systems of Eastern India – an Approach Integrating Socio-economic and Crop Management Factors**

**Authors:** Somsubhra Chakraborty^a,b^, Rupak Goswami^b^, Hirak Banerjee^c^, Sudarshan Dutta^d*^, Kaushik Majumdar^d^, Bin Li^e^, M.L. Jat^f^

*^a^Agricultural and Food Engineering Department, IIT Kharagpur, India-721302*

*^b^IRDM Faculty Centre, RKMVERI, Kolkata-700103, India*

*^c^Regional Research Station (CSZ), BCKV, Kakdwip-743347, India*

*^d^African Plant Nutrition Institute, Lot 660, Hay Moulay Rachid, Benguérir- Morocco.*

*^e^Department of Experimental Statistics, Louisiana State University, LA-70803, USA.*

*^f^International Maize and Wheat Improvement Center (CIMMYT), New Delhi – 110012*

**Corresponding author:**

Dr. Sudarshan Dutta, PhD

Scientist,

African Plant Nutrition Institute

Lot 660, Hay Moulay Rachid, Benguérir- Morocco

Email: S.DUTTA@apni.net.

***Soil Spectral Characterization***

In the laboratory, all 180 samples were scanned using a field portable ASD AgriSpec®VisNIR spectroradiometer (Analytical Spectral Devices, CO, USA) with a spectral range of 350 to 2500 nm. About 30 g of each sample was placed into a Duroplan® borosilicate optical-glass Petri dish and scanned from below using a muglamp with a tungsten quartz halogen light source (Analytical Spectral Devices, CO, USA). Each sample was scanned four times with a 90°rotation between successive scans to obtain an average spectral curve. A spectralon panel with 99% reflectance was used at every five samples to optimize and white reference the spectroradiometer.

***Preprocessing of Spectral Data***

Derivative spectroscopy was used to preprocess soil spectra preceding model analysis. Derivative spectra remove the baseline shift arising from detector inconsistencies, albedo, and sample handling (Demetriades-Shah et al., 1990). If a spectrum is expressed as reflectance, R, as a function of wavelength, λ, the derivative spectra are calculated using Eq. 1, 2, and 3:

Zero order, R=f(λ) (1)

First order, dR/dλ= f’(λ) (2)

Second order, d^2^R/dλ^2^=f’’(λ) (3)

Raw reflectance spectra were processed via a statistical analysis software package, R version 2.11.0 (Chakraborty et al., 2012) using custom ‘R’ routines. These routines involved (i) a parabolic splice to correct for “gaps” between detectors, (ii) averaging replicate spectra, (iii) fitting a weighted (inverse measurement variance) smoothing spline to each spectra with direct extraction of smoothed reflectance, and (iv) 1^st^-derivatives at 10-nm intervals. The 10-nm averaged 1^st^-derivatives spectra were individually combined with the laboratory measured soil parameters. These processed data were used for subsequent PCA analysis.

***Random Forest***

Random Forest (RF) (Breiman, 2001) is an ensemble model consisting of several C&RT, each of which is constructed as follows: (1) first, a bootstrap sample is taken from the original training samples; and (2) then tree is build based on the bootstrapped data. At each split, the candidate set of variables is a random subset of all the variables. For classification, the response is estimated to be the majority vote of predictions from all the trees involved in the forest. For regression, the response is estimated to be the average of predictions from all the trees involved in the forest. Maize yield (t ha^-1^) was transformed *a priori* into discrete classes [1^st^ quartile (Q_1_), 2^nd^ quartile (Q_2_), 3^rd^ Quartile (Q_3_), and 4^th^ quartile (Q_4_)] for classification purpose. Notably, the RF produced the relative variable importance where the influence of a variable was calculated by the number of times it was selected for splitting, weighted by the split-criterion (the Gini impurity criterion) improvement to the model after splitting, and then averaged over all trees. The relative variable importance was then scaled to make the sum add up to 100, with a larger value indicating a stronger influence on the output variable. Moreover, we plotted the RF partial dependence function which summarizes the effect of predictors on the probability of occurrence in response variable after accounting for the average effect of all other predictors.

***Support Vector Machine classification***

Support vector regression (SVR) and support vector machine (SVM) are two well-known support vector methods (Vapnik, 1995). As implied by its name, the solution of the support vector methods, which can be solved through quadric programming, often depends on a small subset of samples in the data, which are called support vectors. The support vector methods have demonstrated superior performances and can be easily extended to nonlinearly transformed feature space via a technique called the “*kernel trick”*. Subsequently, the linear SVM is applied on that high-dimensional space. Although the boundary for linear SVM on that high-dimensional space is linear, when it projects back to its original space, it becomes nonlinear. However, since its solution utilizes all the variables without discrimination, the standard support vector methods suffer from the presence of redundant variables (Guyon et al., 2002). In present study, we used the gaussian kernel (also called radial basis kernel) for the SVM.

***Artificial Neural Network***

We further used artificial neural network (ANN) to test whether it can further improve classification results (Ripley, 1996). Nonlinear ANNs are very flexible and sophisticated modeling techniques competent in modeling complex functions. Automated Network Search (ANS) was used for designing a number of networks to solve the problem and then select those networks that best represent the relationship between the input and target variables (i.e., those networks that attain the highest correlation between the targets and the outputs of the neural network). Only multilayer preceptron (MLP) network which is a widely used ANN model was trained. In general, MLP contains an input layer, hidden layers, and an output layer (Wu et al., 2013) while each layer contains nodes that are connected with a certain weight to every node in the following layer. Excluding the input nodes, each node resembles a processing element by a nonlinear activation function like sigmoid function that allows the network to calculate complex nonlinear problems. For network training, MLP employs the 'back propagation' technique to change connection weights after each data is processed as passing through the nodes in the network in order to decrease the error in the output than the expected result. The training process stops automatically when the generalization stops improving with no further decrease in the errors of cross-validation sample. The hidden-input (Hji) and hidden-output (Ojk) connection weights are saved and used to quantify the relative contribution (RC) of input variables to the predictive output of MLP models using Eq. 4 (Garson, 1991):


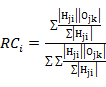
 (4)

The ANN was executed in the open source data mining package WEKA, available athttp://www.cs.waikato.ac.nz/ml/weka/. We optimized MLP parameters using ‘CVParameterSelection’ module in WEKA. Note that RF, SVM, and ANN were applied on the whole dataset and further applied on a split of data (135 training 75% and 44 test 25%).

**REFERENCES**

Chakraborty S, Weindorf DC, Zhu Y, Li B, Morgan CLS, Ge Y, Galbraith J (2012) Spectral reflectance variability from soil physicochemical properties in oil contaminated soils. Geoderma 177-178:80-89.

Demetriades-Shah TH, Steven MD, Clark JA (1990) High-resolution derivative spectra in remote sensing. Remote Sensing of Environ 33:55–64.

Garson GD (1991) Interpreting neural-network connection weights, AI expert 6:46-51.

Guyon I, Weston J, Barnhill S, Vapnik V (2002) Gene selection for cancer classification using SVM. Machine Learning 46:389–422.

Ripley BD (1996) Pattern Recognition and Neural Networks. Cambridge University Press.

Vapnik V (1995) The Nature of Statistical Learning Theory. Springer, NY.

Wu G, Kechavarzi C, Li X, Wu S, Pollard JT, Sui H, Coulon F (2013) Machine learning models for predicting PAHs bioavailability in compost amended soils. Chem Engn J 223:747-754.
